# Determinants of elevated healthcare utilization in patients with COPD

**DOI:** 10.1186/1465-9921-12-7

**Published:** 2011-01-13

**Authors:** Tzahit Simon-Tuval, Steven M Scharf, Nimrod Maimon, Barbara J Bernhard-Scharf, Haim Reuveni, Ariel Tarasiuk

**Affiliations:** 1 Department of Health Systems Management, Guilford Glazer Faculty of Business and Management, Ben-Gurion University, Beer-Sheva, Israel; 2 Division of Pulmonary and Critical Care, University of Maryland, Baltimore, MD, USA; 3 Faculty of Health Sciences, Ben-Gurion University, Beer-Sheva, Israel; 4 Mt. Washington Pediatric Hospital, Baltimore, MD, USA

## Abstract

**Background:**

Chronic obstructive pulmonary disease (COPD) imparts a substantial economic burden on western health systems. Our objective was to analyze the determinants of elevated healthcare utilization among patients with COPD in a single-payer health system.

**Methods:**

Three-hundred eighty-nine adults with COPD were matched 1:3 to controls by age, gender and area of residency. Total healthcare cost 5 years prior recruitment and presence of comorbidities were obtained from a computerized database. Health related quality of life (HRQoL) indices were obtained using validated questionnaires among a subsample of 177 patients.

**Results:**

Healthcare utilization was 3.4-fold higher among COPD patients compared with controls (p < 0.001). The "most-costly" upper 25% of COPD patients (n = 98) consumed 63% of all costs. Multivariate analysis revealed that independent determinants of being in the "most costly" group were (OR; 95% CI): age-adjusted Charlson Comorbidity Index (1.09; 1.01 - 1.2), history of: myocardial infarct (2.87; 1.5 - 5.5), congestive heart failure (3.52; 1.9 - 6.4), mild liver disease (3.83; 1.3 - 11.2) and diabetes (2.02; 1.1 - 3.6). Bivariate analysis revealed that cost increased as HRQoL declined and severity of airflow obstruction increased but these were not independent determinants in a multivariate analysis.

**Conclusion:**

Comorbidity burden determines elevated utilization for COPD patients. Decision makers should prioritize scarce health care resources to a better care management of the "most costly" patients.

## Background

Chronic obstructive pulmonary disease (COPD) is a common respiratory disease affecting more than 10% of adults aged ≥40 yrs [1]. COPD is a leading cause of mortality worldwide [2] and it imparts a substantial economic burden on western health systems [2,3]. It is often accompanied by exacerbations of respiratory symptoms requiring hospitalization [4,5], and therefore is associated with increased health care utilization [1,6,7]. Difference in healthcare cost estimates may stem from differences in payment schemes applied in health system [8]. These in turn may be related to differences in availability and practice patterns. To date, few studies have been conducted in single-payer systems in which availability of resources and practice mandates are uniform.

Concomitant comorbidities among COPD patients are associated with elevated healthcare costs [9,10]. These include other major system diseases such as cardiac, liver, and endocrine disorders such as diabetes. In addition, comorbidities that could influence health costs include sleep disorders such as obstructive sleep apnea (OSA) and insomnia [11,12].

In the present study, we analyzed the determinants of health care utilization, incorporating measures of sleep quality, general and disease specific health related quality of life (HRQoL) and comorbidity burden in a single-payer health system. We hypothesized that HRQoL, sleep disturbances, and comorbidity burden determine elevation of health care utilization in COPD patients.

## Methods

### Setting

A cross-sectional observational study was conducted at the Pulmonary Clinic of the Soroka University Medical Center, a tertiary care referral center with a catchment population of approximately 550,000. Ninety-five percent of patients in this clinic are enrollees of the Clalit Health Services (CHS), the largest health maintenance organization in Israel. The study was approved by the Institutional Ethics Committee (approval number 10283) as well as the committee of Clalit Health Services for extracting data from the database.

### Patients

From March 2009 through December 2009, we prospectively recruited patients (n = 389) attending routine clinic appointments who met the following criteria: 1) enrollees of CHS, 2) age >35, 3) smoking history of ≥ 10 pack-years, 4) pulmonologist-diagnosed COPD. Exclusion criteria were: 1) other major pulmonary diagnoses, 2) concomitant disease expected to shorten life span to <3 years (determined from chart review by one of the investigators - NM), 3) exacerbations of COPD and/or hospitalization/urgent care visits within the month prior to recruitment (in order to obtain both clinical and HRQoL indices from stable patients). As a part of another study on HRQoL in COPD [13,14], in a subset of 177 patients, data were collected on HRQoL as well as measures of sleep quality. As control subjects, patients without COPD were randomly selected from the database of CHS enrollees, matched 1:3 to the COPD patients (n = 1,167) by age, gender, primary-care clinic and area of residency.

### Measures

*Spirometric indices of lung function *[15] were obtained within 6 months prior to the sentinel clinic visit from the patient's medical record at the pulmonary clinic, and included forced vital capacity (FVC) and forced expired volume in one second (FEV_1_). *Disease severity *was staged according to the Global Initiative for Lung Disease (GOLD - 2006) [16], and % predicted FEV_1_. *Demographics *including age, gender, body mass index (BMI), smoking status (current, ex-smoker), and pack-years smoking, were obtained from the patient's medical record at the pulmonary clinic. In the subset of 177 patients, indices of *Health Related Quality of Life *(HRQoL) and sleep quality were obtained using trained interviewers as described in another study of our group [13,14] applying Hebrew translations of four-week recall questionnaires that included: 1) a generic questionnaire, the Health Utilities Index 3 (HUI3); 2) a disease specific questionnaire, the St. George's Respiratory Questionnaire (SGRQ); and 3) the Pittsburgh Sleep Quality Index (PSQI). Among this subset we collected data on socioeconomic status including: income relative to the Israeli average income, years of schooling, employment status and marital status. The presence of *Comorbidities *was obtained from CHS database, using the International Classification of Diseases, Ninth Revision (ICD-9) codes. The age-adjusted Charlson Comorbidity Score with Deyo Modification (CCI) [17] was calculated accordingly. Additionally, we assessed the presence of hypertension, depression, obstructive sleep apnea and pulmonary hypertension (that are not included in CCI and commonly found in COPD patients).

Information regarding annualized health care utilization was obtained for the five year period prior to the end of recruitment period (December 15, 2009) from the CHS financial database [18]. Under the obligatory Israeli National Health Insurance Law, all citizens have equal access to medical services. Physicians are generally paid a capitation fee and thus do not have economic incentive to increase healthcare consumption. Indicators of health care utilization included: hospitalization, emergency department visits, visits to specialists (consultations), surgeries including operative procedures such as cardiac catheterization and heart or spinal column surgeries, diagnostic procedures including CT scans, Ultrasound, MRI and spirometry, and medication according to the WHO classification system [19]. Although patients who had exacerbations of COPD and/or hospitalization/urgent care visits within the month prior to recruitment were excluded, our retrospective analysis over 5 years included patients who experienced exacerbation during these years but the data for the number of exacerbation were not available. Utilization costs estimates were based on a standardized price-list published by the Israeli Ministry of Health in 2009. Medication costs estimates were based on a price-list published by the CHS. All costs are expressed in US dollars ($) with an assumed exchange rate of 3.7 New Israeli Shekels per US dollar.

### Data and Statistical Analysis

Data were analyzed using STATA software (ver 11.0, StataCorp, USA). Non-normally distributed variables were presented as median with 25-75 percentiles unless otherwise specified. Dichotomous indicator values were presented as proportions. Since health care utilization costs are not normally distributed, we stratified our COPD patients cohort into two subgroups [18]- the upper 25% (n = 98) who were the "most costly" patients and the "remaining" 75% (n = 291). Comparison between group medians was done using Mann-Whitney U test, and between proportions using Chi-square test. Regression was done using the least-squares technique. The null hypothesis was rejected at the 5% level.

Significant bivariate predictors of elevated health care utilization were put into a multivariate logistic regression. Independent variables included: age, gender, BMI, disease severity (percent predicted FEV_1_, GOLD class), Comorbidities (number and category), age-adjusted CCI, smoking history (pack-yrs), HUI3, SGRQ, PSQI. In order to examine whether the model has predictive ability we obtained the area under the receiver operating characteristic (ROC) curve.

## Results

Three hundred eighty nine patients with COPD were included in our cohort (median age of 68 and 78% male gender). The non-COPD control subject were similar in age and gender, but had lower comorbidity burden as measured by age-adjusted CCI (4 vs. 7, p < 0.001). The most prevalent diseases (>30% of the population) in this group were hypertension, connective tissue disease and diabetes. As depicted in Table 1, the median annualized cost of health care for the entire COPD cohort (n = 389) was $2200 (25 - 75 percentiles: $1139 - $4934), 3.4 times higher than the non-COPD controls (p < 0.001). This elevated healthcare consumption stemmed mainly from increased utilization of hospitalization, medication and diagnostic procedures.

**Table 1:** Comparison of total cost elements between COPD patients and matched controls.

	control (n = 1167)	COPD (n = 389)	P value*
**Annualized Total Cost**	1634 ± 2480	3823 ± 4794	<0.001
**(US$/person)**	652 (225 - 2013)	2200 (1139 - 4934)	

**Hospitalization**	410 ± 1083	1474 ± 2428	<0.001
Annualized Costs ($US/person)	0 (0 - 352)	615 (106 - 1614)	

**Surgeries**	564 ± 1309	825 ± 1789	<0.001
Annualized Costs ($US/person)	0 (0 - 477)	0 (0 - 675)	

**Diagnostic procedures**	222 ± 256	440 ± 349	<0.001
Annualized Costs ($US/person)	133 (40 - 316)	348 (184 - 607)	

**Consultations**	89 ± 94	201 ± 148	<0.001
Annualized Costs ($US/person)	59 (24 - 124)	171 (88 - 271)	

**Emergency Room Visit**	33 ± 51	56 ± 80	<0.001
Annualized Costs ($US/person)	27 (0 - 55)	29 ( 0 - 59)	

**Medication**	297 ± 1042	790 ± 3031	<0.001
Annualized Costs ($US/person)	104 (28 - 288)	414 (220 - 725)	

Values are presented as mean ± SD and median (25-75 percentiles).

* Mann-Whitney U test.

As demonstrated in Table 2, the subset of 177 COPD patients that were interviewed resembled the entire COPD cohort (n = 389) with regard to demographic characteristics, severity of airflow obstruction, smoking history, comorbidity burden and healthcare costs. The "most costly" COPD patients (n = 98) consumed 63% of all costs and had a median annualized cost of $7692 per patient (25 - 75 percentiles: $6365 - $9892), 4.7 times higher than the remainder (n = 291), whose median annualized cost was $1632 (25 - 75 percentiles: $949 - $2660, p < 0.001). The characteristics of the "most costly" patients compared to the rest of the study population are summarized in Table 3. Compared to the rest of the patients, the "most costly" patients were older had significantly more comorbid conditions and higher age adjusted CCI. The most costly patients had significantly lower percent predicted FEV_1 _than the others but were not different in severity class according to the GOLD criteria. In addition, no significant differences were found between groups in BMI and smoking history (pack-yrs). In the subset of 177 COPD patients, we found no significant difference between the "most costly" patients and the remainders in socioeconomic status and HRQoL indices.

**Table 2:** Comparison of characteristics between entire cohort and the subset group.

Variable	Entire cohort	The subset group	P value
N	389	177	

Age (yrs)*	68 (59 - 77)	67 (60 - 74)	0.25^†^

Males (%)	77.6%	78.0%	0.93^‡^

BMI (kg/m^2^)*	27 (24 - 31)	27 (23 - 30)	0.57^†^

Pack yrs smoking (yrs)*	50 (30 - 80)	40 (28 - 60)	0.07^†^

FEV_1 _(% predicted)*	47 (37 - 60)	46 (36 - 58)	0.49^†^

GOLD class 3 or 4	54.0%	59.9%	0.19^‡^

Age adjusted CCI*	7 (4 - 9)	6 (4 - 9)	0.71^†^

Annualized healthcare cost*	2200 (1139 - 4934)	2312 (1139 - 5519)	0.61^†^

Abbreviations: BMI- body mass index, FEV_1_- forced expired volume in one second (as percent predicted), GOLD- global initiative for chronic obstructive lung disease, CCI- Charlson Comorbidity Index.

* median (25-75 percentiles); ^† ^Mann-Whitney U test; ^‡ ^Chi-square test.

**Table 3:** Characteristics of adult COPD patients (n = 389)- Comparison between the "Most costly" patients and the remainder.

Variable		"Most costly"*	The remainder	P value
N		98	291	

Age (yrs)^†^		70 (65 - 77)	67 (58 - 76)	0.003^‡^

Males (%)		84.7%	75.3%	0.05^§^

BMI (kg/m^2^)^†^		27 (24 - 30)	27 (24 - 31)	0.74^‡^

Pack yrs smoking (yrs)^†^		50 (35 - 80)	50 (29 - 75)	0.10^‡^

FEV_1 _(% predicted)^†^		44 (33 - 56)	49 (38 - 61)	0.03^‡^

GOLD class 3 or 4		62.2%	51.2%	0.06^§^

Number of morbidity conditions^†^		6 (4 - 8)	3 (1 - 4)	<0.001^‡^

Age adjusted CCI^†^		9 (7 - 11)	5 (3 - 8)	<0.001^‡^

Education (yrs of schooling) ^†,‖^		12 (8 - 12)	10 (4 - 12)	0.07^‡^

	Low	83%	80%	

Income^¶,‖^	Average	10%	10%	0.85^§^

	High	7%	10%	

PSQI^†,‖^		13 (7 - 16)	11 (6 - 16)	0.48^‡^

SGRQ^†,‖^		67.7 (43.5 - 77.1)	57.7 (41.8 - 72.3)	0.17^‡^

HUI3^†,‖^		0.6 (0.3 - 0.7)	0.7 (0.3 - 0.8)	0.13^‡^

Abbreviations: BMI- body mass index, FEV_1_- forced expired volume in one second (as percent predicted), GOLD- global initiative for chronic obstructive lung disease, CCI- Charlson Comorbidity Index, PSQI- Pittsburgh sleep quality index, SGRQ- St Georges Respiratory Questionnaire, HUI3- health utilities index mark 3.

* The "most costly" patients are those whose annualized utilization cost was within the upper 25 percentile; ^† ^median (25-75 percentiles); ^‡ ^Mann-Whitney U test; ^§ ^Chi-square test; ^‖ ^This indicator was calculated on the subsample of 177 patients; ^¶ ^Self reported income levels were defined as Low/Average/High relative to the average monthly income ($2,160/month).

The most prevalent comorbidities among the "most costly" patients were: hypertension, myocardial infarct, congestive heart failure and diabetes mellitus (Table 4). These comorbidities are significantly more prevalent in this sub-group compared to the rest of the patients. Connective tissue disease is also a common comorbidity among the "most costly" patients, but its prevalence is not significantly different from that among the rest of the patients. There was no evidence of increased odds for the presence of either OSA or depression/anxiety among the "most costly" group compared to the "remainder".

**Table 4:** Prevalence of comorbidities among the "most costly" patients compared to the remainder.

Comorbidity condition (ICD-9 codes)	Prevalence				
			
	"Most costly"* **(n = 98)**	The remainder (n = 291)	**P value**^†^	OR	95% CI
Hypertension (401 - 405)	74%	54%	<0.001	2.53	1.5 - 4.2

Myocardial infarct (410, 411)	70%	29%	<0.001	5.96	3.6 - 9.9

Connective tissue disease (710, 714, 725)	58%	57%	0.89	1.03	0.6 - 1.6

Congestive heart failure (398, 402, 428)	52%	14%	<0.001	6.81	4.1 - 11.4

Diabetes Mellitus (250)	50%	24%	<0.001	3.10	1.9 - 5.0

Moderate and severe renal disease (403, 404, 580 - 586)	37%	20%	0.001	2.38	1.4 - 3.9

Hemiplegia (342, 434, 436, 437)	29%	13%	<0.001	2.66	1.5 - 4.6

Peripheral vascular disease (440 - 447)	24%	13%	0.01	2.09	1.2 - 3.7

Cerebrovascular disease (430 - 433, 435)	23%	12%	0.01	2.17	1.2 - 3.9

Depression/Anxiety (296.2, 296.3, 311)	23%	15%	0.06	1.72	1.0 - 3.0

Peptic ulcer (531 - 534)	22%	15%	0.08	1.67	0.9 - 3.0

Diabetes Mellitus with organ damage (250.4 - 250.7)	21%	9%	0.001	2.78	1.5 - 5.2

Pulmonary hypertension (415, 416)	20%	7%	<0.001	3.67	1.9 - 7.2

Any tumor (140 - 195)	17%	10%	0.05	1.90	1.0 - 3.6

Obstructive sleep apnea (780.51, 780.53)	16%	9%	0.06	1.89	1.0 - 3.7

Mild liver disease (571, 573)	8%	3%	0.02	3.14	1.1 - 8.6

Moderate or severe liver disease (070, 570, 572)	7%	2%	0.01	4.40	1.4 - 14.2

Dementia (290, 291, 294)	3%	2%	0.42	1.81	0.4 - 7.7

Lymphoma (200, 202, 203)	1%	0%	0.42	2.99	0.2 - 48.4

Metastatic solid tumor (196 - 199)	1%	2%	0.63	0.59	0.1 - 5.1

Leukemia (204-208)	1%	1%	0.74	1.49	0.1-16.7

None of the patients in this cohort had AIDS.

* The "most costly" patients are those whose annualized utilization cost was within the upper 25 percentile; ^† ^Chi-square test.

All health care utilization components were significantly greater among the "most costly" patient compared to the rest of the patients (Table 5). Predominant components of patients' health care utilization are hospitalization, surgeries, diagnostic procedures and medication. Seventy-three percents of the surgeries cost among the "most costly" patients were related to heart disease, i.e. cardiac catheterization, heart surgery and implantation of a pace-maker.

**Table 5:** Comparison of total cost elements between the "Most costly" COPD patients and the remainder.

	"Most costly"*(n = 98)	The remainder (n = 291)	**P value**^†^
**Annualized Total Cost**	9681 ± 6475	1899 ± 1185	<0.001
**(US$/person)**	7692 (6365 - 9892)	1632 (949 - 2660)	

**Hospitalization**	4129 ± 3549	607 ± 745	<0.001
Annualized Costs ($US/person)	3147 (1567 - 6061)	352 (0 - 875)	

Annualized Days/person	10.4 ± 10.7	1.4 ± 1.7	<0.001
	7.4 (3.6 - 13.8)	0.8 (0 - 2.0)	

Annualized	2.4 ± 1.9	0.5 ± 0.6	<0.001
Admissions/person	1.9 (1.0 - 3.0)	0.2 (0 - 0.6)	

Average Length	4.3 ± 2.4	3.4 ± 2.2	<0.001
Days/Admission	3.7 (3.0 - 4.9)	3.0 (2.0 - 4.0)	

**Surgeries**	2557 ± 2817	268 ± 565	<0.001
Annualized Costs ($US/person)	1991 (477 - 4166)	0 (0 - 238)	

Annualized	0.4 ± 0.3	0.1 ± 0.1	<0.001
Number/person	0.4 (0.2 - 0.6)	0 (0 - 0.2)	

**Diagnostic procedures**	676 ± 444	357 ± 264	<0.001
Annualized Costs ($US/person)	631 (339 - 849)	289 (161 - 506)	

Annualized	8.6 ± 4.9	5.5 ± 3.4	<0.001
Number/person	7.4 (5.2 - 12.2)	4.8 (3.0 - 7.2)	

**Consultations**	278 ± 180	174 ± 124	<0.001
Annualized Costs ($US/person)	256 (141 - 381)	157 (77 - 240)	

Annualized	9.1 ± 6.0	5.7 ± 4.0	<0.001
Visits/person	8.2 (4.4 - 12.6)	5.0 (2.4 - 8.0)	

**Emergency Room Visit**	108 ± 121	40 ± 51	<0.001
Annualized Costs ($US/person)	59 (29 - 147)	29 ( 0 - 59)	

Annualized	0.7 ± 0.8	0.3 ± 0.3	<0.001
Visits/person	0.4 (0.2 - 1.0)	0.2 (0 - 0.4)	

**Medication**	1856 ± 5855	432 ± 327	<0.001
Annualized Costs ($US/person)	709 (420 - 1171)	340 (191 - 636)	

Annualized Number of	109.8 ± 51.3	63.8 ± 45.4	<0.001
Prescriptions/person	104.1 (72.4 - 139.2)	53.0 (30.0 - 93.6)	

Values are presented as mean ± SD and median (25-75 percentiles).

* The "most costly" patients are those whose annualized utilization cost was within the upper 25 percentile.

^† ^Mann-Whitney U test.

The median annualized medication cost for the "most costly" patient was 2.1 times higher than among the remainder. Table 6 depicts drug utilization according to drug classification. The most frequently used drugs were those categorized as respiratory, cardiovascular, alimentary tract and metabolism. The consumption of analgesics, psycholeptics and psychoanaleptics was low but significantly higher among the "most costly" patients.

**Table 6:** Comparison of the annualized medication cost ($US/person) between the "Most costly" COPD patients and the remainder.

Pharmacological classification	"Most costly"* **(n = 98)**	The remainder (n = 291)	**P value**^†^
Total	**709 (420 - 1171)**	**340 (191 - 636)**	

R- Respiratory System	242 (118 - 400)	154 (56 - 330)	0.01

C- Cardiovascular System	78 (24 - 151)	15 (1 - 61)	<0.001

A- Alimentary Tract & Metabolism	48 (18 - 103)	9 (2 - 35)	<0.001

J- General Antiinfectives for Systemic Use	17 (12 - 31)	12 (6 - 17)	<0.001

B- Blood and Blood Forming Organs	14 (3 - 42)	1 (0 - 5)	<0.001

N- Nervous System	11 (3 - 40)	4 (1 - 16)	<0.001

N02- Analgesics	3.5 (1.5 - 8.5)	1.3 ( 0.3 - 3.5)	<0.001

N05, N06- Psycholeptics, Psychoanaleptics	0.8 (0 - 8.6)	0.2 (0 - 3.7)	0.02

M- Musculo-Skeletal System	5 (2 - 25)	4 (1 - 12)	0.01

H- Systemic Hormonal Preparations, Excluding Sex Hormones	4 (1 - 11)	1 (0 - 4)	<0.001

D- Dermatologicals	4 (1 - 10)	2 (0 - 7)	0.002

G- Genitourinary System & Sex Hormones	3 (0 - 69)	1 (0 - 17)	0.05

S- Sensory Organs	3 (1 - 11)	1 (0 - 5)	<0.001

L- Antineoplastic and Immunomodulating Agents	0 (0 - 0)	0 (0 - 0)	0.001

P- Antiparasitic Products, Insecticides and Repellants	0 (0 - 0)	0 (0 - 0)	<0.001

Values are presented as median (25 - 75 percentiles).

* The "most costly" patients are those whose annualized utilization cost was within the upper 25 percentile.

^† ^Mann-Whitney U test.

In the subset of 177 patients in whom we collected HRQoL data, bivariate regression between HRQoL indices (as measured by PSQI, SGRQ and HUI3) and annualized healthcare cost revealed that cost increased as HRQoL declined for all measures (PSQI: slope = 85.9, p = 0.04, adjusted R-squared = 0.02; SGRQ: slope = 22.7, p = 0.03, adjusted R-squared = 0.02; HUI3: slope = -1656.2, p = 0.003, adjusted R-squared = 0.04). However, these indices did not remain as independent predictors of cost in the presence of comorbidity burden in the multivariate model.

Multivariate logistic regression, adjusting for age, FEV_1 _and BMI, revealed that comorbidity burden (as measured by age-adjusted CCI) and the presence of myocardial infarct, congestive heart failure, mild liver disease and diabetes mellitus were independent determinants for being "most costly" COPD patients (Table 7). The area under the ROC curve was 0.82, implying that the model has strong predictive power.

**Table 7:** Determinants of the upper quarter most costly COPD patients.

	Bivariate analysis (n = 389)			Multivariate analysis* (n = 388)		
	**OR**	**95% CI**	**P value**	**OR**	**95% CI**	**P value**

Age (year +1)	1.03	1.0 - 1.1	0.001	NI		

FEV_1_%	0.99	0.97 - 1.0	0.07	0.99	0.97 - 1.01	0.25

BMI (+1 Kg/m^2^)	1.01	1.0 - 1.1	0.50	0.98	0.93 - 1.04	0.54

Age adjusted CCI	1.27	1.2 - 1.4	<0.001	1.09	1.01 - 1.19	0.04

Myocardial infarct	5.96	3.6 - 9.9	<0.001	2.87	1.5 - 5.5	0.001

Congestive heart failure	6.81	4.1 - 11.4	<0.001	3.52	1.9 - 6.4	<0.001

Mild liver disease	3.14	1.1 - 8.6	0.03	3.83	1.3 - 11.2	0.02

Diabetes mellitus	3.10	1.9 - 5.0	<0.001	2.02	1.1 - 3.6	0.02

Abbreviations: FEV_1_- forced expired volume in one second (as percent predicted), BMI- body mass index, CCI- Charlson Comorbidity Index, NI- not included (due to insignificance).

* Area under ROC curve equals 0.82.

## Discussion

In this study, we have provided additional evidence of higher healthcare cost in COPD patients compared to matched non-COPD controls. In addition, our results demonstrated that the odds of being among the most costly COPD patient were associated with comorbidity burden as well as specific comorbidities, namely: concomitant heart disease (myocardial infarct, congestive heart failure), mild liver disease and diabetes mellitus. Severity of airflow obstruction and HRQoL indices were not independent determinants of increased health care utilization. The following discussion considers these results in light of the currently available literature.

### Elevated healthcare utilization

COPD patients consumed 3.4 times higher healthcare resources compared to controls. Since control subjects were randomly matched 1:3 to COPD cohort by age, it can be assumed that most characteristics are typical to this age range except for the elevated burden associated with COPD. Similar trends have been found previously [6,7,10]. Two studies conducted among Medicaid enrollees older than 45 in Maryland [7,10] showed that that COPD patients consumed 1.33 time greater healthcare resources and had 1.8 times greater adjusted average number of inpatient claims compared to controls. Mapel et al. [6] found that healthcare utilization among COPD patients in New Mexico was approximately twice that of age and gender matched controls. Our results extend these previous ones showing that the same trends apply in a single-payer health system including various socioeconomic groups and extended age range. Our estimates may differ from those observed in other countries [1] due to variety of factors, among which the most important are: patients' selection method, differences in health system's payment schemes and in price-lists.

### The effect of comorbidities

Each increase in age-adjusted CCI increased the odds of being a "most costly" COPD patient. The specific comorbidities predicting being in the "most costly" group were myocardial infarction, congestive heart failure, mild liver disease and diabetes. In the study of Lin and colleagues [10], determinants of health care utilization in COPD patients compared with others were diabetes with organ damage, peptic ulcer, congestive heart failure and mild liver disease. Thus, a number of the determinants of health care utilization in COPD patients compared with non-COPD patients also determine elevated health care costs within the COPD patient group. Even though our patients are from an extended age range with an older mean compared those of Lin et al, in both groups heart disease, diabetes and liver disease figure prominently as important comorbidities increasing health care utilization.

The connection between cardiovascular disease and COPD has been reported previously [6,20,21]. In our sample, these findings were reinforced by our findings that both congestive heart failure and myocardial infarction were independent predictors of being in the "most costly" group. Further, our results revealed increased utilization of cardiovascular drugs and increased costs related to cardiac surgeries among the "most costly" patients. Thus, it appears that managing care of COPD with concomitant cardiovascular disease should be one of the major foci for intervention in patients with COPD.

The presence of mild liver disease increased the odds of belonging to the "most costly" COPD patient. Although there is no single pathogenetic mechanism involved, chronic liver dysfunction may cause pulmonary manifestations because of alterations in the production or clearance of circulating cytokines and other mediators [22]. Further, this association may be related to the effect of smoking that is an important risk factor for COPD and is commonly reported by patients with advanced liver disease.

The co-presence of diabetes was an additional predictor for increased health care utilization. This result is consistent with previous studies showing that diabetes is a predictor of longer hospitalizations and adverse clinical outcomes in patients with acute exacerbations of COPD [5,23]. In this regard, increased length of stay was a component of increased health care utilization for the "most costly" patients. From the database, we cannot determine precisely whether the "most costly" patients' hospitalizations were longer due to poor glucose control, but this could have been one contributor.

COPD is associated with significantly higher risk of having anxiety/depressive symptoms [24]. Recent studies had demonstrated that these symptoms among COPD patients were associated with an increased risk of COPD exacerbations and hospitalization [25,26]. Hence, we expected that patients with COPD with elevated health care utilization would have been more likely to be diagnosed with anxiety and/or depression and would have thus consumed drugs to treat these conditions. Although our study did not include measures of anxiety and depression, we found that there was no significant difference between the most costly patient and the remainder in the prevalence of anxiety and depression, and the utilization of psychoactive drugs was low. This result may stem from the study population size, the willingness of physicians to address anxiety/depression in their COPD patients, or local practice patterns and needs further examination.

We found that the presence of concurrent OSA was not an independent predictor of elevated healthcare utilization. These results appear to be in conflict with the results of Shaya and colleagues [12] showing that the presence of OSA adds additional economic burden on beneficiaries who already have COPD. The discrepancy may relate to the fact that in neither study were attempts made to assess the true prevalence of OSA in COPD patients. However, the proportion of patients with OSA in the "most costly" group was greater in our study, but did not reach statistical significance. The sample of Shaya et al was considerably larger than ours, and it is possible that with larger numbers, our conclusions would have been the same as those of Shaya et al.

### The effect of airflow obstruction

Interestingly, the severity of airflow obstruction was not an independent predictor of health care cost on multivariate analysis. It appears that once a patient has COPD, other factors, primarily comorbidities, determine health care utilization cost. Thus, the physiological impairment, while predicting mortality [5,20], does not predict health care utilization independently of other comorbid conditions.

### The effect of HRQoL

Our study demonstrated that as indices of health related quality of life (HRQoL) decline, annualized healthcare utilization increases. However, when the burden of specific comorbidities was taken into account, HRQoL *per se *was not a predictor of utilization. According to Sin and colleagues [20], the presence of comorbidities was associated with higher scores (implying worse HRQoL) on St George's Respiratory Questionnaire (SGRQ). Similar trends were found in another recent study of our group [13,14]. Thus, it is most likely that HRQoL reflected the comorbidity burden.

## Limitations

There are a number of limitations in the present study. First, our database lacked information about reasons leading to hospitalizations (discharge diagnoses). Second, estimates of health care utilization may not be applicable to other health care systems, as practice patterns and costs may differ. Third, sleep studies were not part of our study protocol and the presence of OSA was assessed using the patients' medical records. Finally, over-fitting of our multivariate regression analysis is a potential concern. We have attempted to be parsimonious regarding the number of explanatory variables, and have tried to include those that appeared to be biologically and clinically relevant for COPD patients. These included age, degree of airflow obstruction, body mass, and overall and specific comorbidity burden. Further study is needed to substantiate our results.

## Conclusions

Compared to controls, COPD patients consume 3.4 times higher healthcare resources. The "most costly" patients with COPD consumed 63% of all costs and their median annualized cost was 4.7 times higher compared to the remainder. Comorbidity burden, not the severity of airflow obstruction and HRQoL indices, is the most important independent predictor of increased healthcare cost. Care management of costly patients with COPD should be the focus of health care decision makers, whose aim is to efficiently allocate scarce resources. Further study is needed to evaluate the cost effectiveness of interventions directed at "costly" COPD patient with specific comorbidities to improve their health outcomes.

## Abbreviations

BMI: body mass index; CCI: Charlson comorbidity index; CHS: Clalit Health Services; COPD: chronic obstructive pulmonary disease; FEV_1_: Forced expired volume in one second; GOLD: Global initiative for obstructive lung disease; HRQoL: Health related quality of life; HUI: Health Utilities Index; OSA: Obstructive sleep apnea; PSQI: Pittsburgh Sleep Quality Index; SGRQ: St Georges Respiratory Questionnaire.

## Competing interests

The authors declare that they have no competing interests.

## Authors' contributions

Conception and design: TST, SMS, HR, AT; Analysis and interpretation of the data: TST, SMS; Drafting of the article: TST, SMS; Critical revision of the article for important intellectual content: TST, SMS, HR, AT; Statistical expertise: TST, SMS, BJBS; Administrative, technical, or logistic support: TST, NM, BJBS, AT; All authors have read and approved the final manuscript.
